# Attitudes of staff towards smoke-free environments in psychiatric hospitals in Germany

**DOI:** 10.18332/tid/152252

**Published:** 2022-09-05

**Authors:** Deniz Cerci

**Affiliations:** 1 Klinik fur Forensische Psychiatrie, Universitatsmedizin Rostock, Rostock, Deutschland

**Keywords:** smoke-free environment, tobacco control, staff attitudes, psychiatry, mental health

## Abstract

**INTRODUCTION:**

Smoke-free environments have already been successfully introduced in hospitals world-wide. But despite convincing evidence of their success, many countries still struggle to make the necessary changes. Not only is the smoking prevalence higher amongst people with mental health problems and staff working in psychiatric units, but employees in psychiatry often resist the implementation of smoke-free policies. This study explores staff attitudes towards smoke-free environments in psychiatric hospitals in Germany and tries to identify barriers and opportunities for implementation.

**METHODS:**

This cross-sectional online survey was carried out at eight psychiatric units of the state-owned healthcare company *Vivantes Netzwerk für Gesundheit GmbH* in Berlin, Germany, in 2019. A total of 448 members of staff were surveyed on their views towards creating a smoke-free environment in their workplace.

**RESULTS:**

Psychiatric staff present contradictory attitudes towards implementing smoke-free regulations. On the one hand, a majority recognizes the need for smoke-free environments as they promote physical well-being of staff and patients. On the other hand, a majority opposes comprehensive restrictions like a complete smoking ban. Smokers are more likely than non-smokers to resist restrictive measures and show a tendency to only support those measures which they deem unlikely to affect their own smoking habits.

**CONCLUSIONS:**

The contradictory attitudes towards implementing smoke-free regulations present an entry point to elicit behavior change and a shift in attitudes, for example in staff training on smoke-free environments. Staff who smoke, in particular, should be motivated to reflect on the contradiction that is presented by their private smoking behavior and their role as healthcare professionals.

## INTRODUCTION

A growing body of evidence shows that legislative measures to promote smoke-free environments have positive effects on public health, amongst them a reduction in respiratory symptoms in the workplace and lower admission rates for myocardial infarction^1-3^. This also applies to healthcare settings such as hospitals, where smoke-free environments have been introduced across the globe^4-6^.

In psychiatric hospitals, however, measures to discourage or ban smoking are often difficult to implement^7,8^. For decades, the tobacco industry targeted its marketing for people with mental health problems, suggesting that smoking has a calming effect on the mind. This strategy, which included utilizing advertising spaces in the direct vicinity of mental health facilities and delivering free cigarettes to psychiatric hospitals, has shaped the attitudes not only of patients but also of professionals working in these settings^9,10^.

Members of staff often assume that smoking bans cause disruptions in clinical care^11^. However, the implementation of a smoke-free environment in psychiatry is normally less burdensome than staff initially fear. Concerns about increased aggression, discharges against medical advice and higher seclusion rates have been shown to be unfounded^2,12,13^. Instead, smoke-free environments not only improve the physical well-being of psychiatric patients and staff, but also achieve better long-term mental health outcomes and improve the patients’ quality of life^2,14,15^. Mental health does not worsen as a result of quitting smoking, and smoking cessation is likely to be associated with improvements in mental health^16^. Furthermore, the implementation of smoke-free policies can bring about a more patient and recovery focused attitude in staff^2^. However, there seems to be a fundamental mismatch between what staff in psychiatric hospitals assume about the negative effects of smoke-free environments and the scientific evidence on its positive effects.

Consequently, it has become an emerging field of study to investigate what staff think and believe about smoke-free measures and why they resist them^12,17-19^. Studies have shown that individual smoking behavior among staff is associated with smoking-related knowledge and permissive attitudes and practices^20,21^, but only little is known about this field in the German health system. However, investigating staff resistance in Germany seems to be particularly urgent, as implementing smoke-free environments here has been comparatively weak. In a recently published comparison of tobacco control measures in Europe, Germany takes the last place^22^. It is the only country in the EU where billboard advertising for tobacco is still allowed^23^. Smoking is forbidden in public transport, but in many bars and restaurants, designated smoking areas still exist^24^. In healthcare environments, complete and even partial smoking bans and rules regarding smoking are commonly disregarded by the public^25^.

This study closes this gap by providing a comprehensive exploration of staff attitudes regarding the implementation of smoke-free environments in psychiatric units on eight hospital sites in Berlin, the largest study of its kind in Germany. It was based on the hypothesis that support for measures to implement a smoke-free environment in psychiatry is significantly lower amongst staff who smoke versus staff who do not smoke.

## METHODS

*Vivantes Netzwerk für Gesundheit GmbH* is a state-owned healthcare company in Berlin which runs ten hospitals with eight psychiatric units and a number of other healthcare and nursing facilities for inpatients and outpatients. Although smoking is officially not permitted, there are ample exceptions. Patients, visitors and staff can smoke within designated smoking areas on hospital grounds. Psychiatric inpatients, in particular, often have access to additional smoking rooms on the ward and further exceptions are regularly made under special circumstances, e.g. patients in seclusion.

In this study, all members of staff working in psychiatric units were invited to complete an online questionnaire covering different topics in relation to smoking cessation and the creation of a smoke-free environment. The survey was promoted via different channels, including the company newsletter and promotional material and by direct contact of supervising members of staff. It was carried out from 1 October 2019 to 15 November 2019 using the software Lime Survey and participants were entered into a prize draw for vouchers for a department store in Berlin. Participation in the survey was voluntary and all answers were given anonymously. Data analysis was carried out using the software IBM SPSS Statistics 26 and Microsoft Excel 2016. Ethics approval was granted by *Hochschule Neubrandenburg.*

The questionnaire was designed by the author and based on similar surveys found in the literature^12,17-19^. Completion of all questions took less than 10 minutes. The questions in the survey explored, amongst other smoking-related questions, attitudes towards a smoke-free environment in psychiatry and demographic data including smoking status of the participant.

## RESULTS

In all, 448 out of a total of 1706 members of psychiatric staff completed the questionnaire in full, yielding a response rate of 26.3%.

Demographic data showed an age range from below 20 to over 59 years with approximately a third of participants aged 30–39 years (Table 1). More than two-thirds of participants identified as female. One in two participants worked as a nurse. Less than one in five participants were doctors and a slightly lower proportion worked as medico-therapeutic staff (e.g. occupational therapists, physiotherapists, music therapists, dance therapists and other professionals).

**Table 1:** Demographic characteristics and smoking status of participating members of staff in eight psychiatric units, Berlin, Germany, 2019 (N=448)

*Characteristics*	Total % (n)*	*Current non-smokers % (n)*	*Current smokers % (n)*
**Age** (years)			
<20	0.7 (3)	66.7 (2)	33.3 (1)
20–29	17.9 (80)	61.5 (48)	38.5 (30)
30–39	34.4 (154)	60.4 (93)	39.6 (61)
40–49	20.5 (92)	64.4 (58)	35.6 (32)
50–59	17.9 (80)	72.7 (56)	27.3 (21)
>59	7.4 (33)	81.8 (27)	18.2 (6)
**Gender**			
Female	68.8 (308)	66.9 (202)	33.1 (100)
Male	28.1 (126)	60.5 (75)	39.5 (49)
Diverse	1.3 (6)	83.3 (5)	16.7 (1)
**Professional group**			
Doctor	16.1 (72)	72.2 (52)	27.8 (20)
Nurse	51.6 (231)	55.6 (125)	44.4 (100)
Medico-therapeutic staff**	18.1 (81)	81.5 (66)	18.5 (15)
Administrative staff	2.9 (13)	84.6 (11)	15.4 (2)
Other	9.4 (42)	65.9 (27)	34.1 (14)
**Working in substance-abuse services**			
Yes	32.4 (145)	62.5 (90)	37.5 (54)
No	65.0 (291)	67.0 (191)	33.0 (94)
**Smoking status**			
Daily smokers	22.1 (99)		
Occasional smokers (less frequent than daily)	11.8 (53)		
Ex-smokers	23.2 (104)		
Non-smokers	40.4 (181)		

* The option ‘no answer’ provided in the questionnaire is not listed separately in the tables, which explains why the values do not add up to 100%.

** Medico-therapeutic staff includes occupational therapists, physiotherapists, music therapists, dance therapists and other professionals.

Approximately a third worked in substance-abuse services; 63.6% did not smoke or had given up smoking completely. A third smoked daily or occasionally. The highest percentage of current smokers were aged 30–39 years (39.6%). The smoking prevalence in male hospital staff was slightly higher than in female staff. Amongst nursing staff, there were roughly twice as many current smokers as amongst other professional groups. In substance-abuse services, staff smoking rates were slightly higher than in other psychiatric facilities.

Staff were asked for their views towards a smoke-free environment (Table 2) and if they supported or opposed specific measures in the process of implementation (Table 3).

**Table 2:** Attitudes towards a smoke-free environment in participating members of staff in eight psychiatric units, Berlin, Germany, 2019 (N=448)

*Question*	*Reply*	*Total % (n)*	*Current non-smokers % (n)*	*Current smokers % (n)*	*Comparison statistics*
Do you feel bothered by people who smoke in your everyday work?	Yes	35.7 (160)	52 (143)	10.2 (15)	χ²=71.45
	No	60.9 (273)	48 (132)	89.8 (132)	df=1
					p=0.00
Do you believe that medical and therapeutic staff should serve as role models in relation to smoking?	Yes	65.2 (292)	80.4 (217)	51.1 (72)	χ²=38.12
	No	28.4 (127)	19.6 (53)	48.9 (69)	df=1
					p=0.00
Should staff be allowed to smoke in the presence of patients?	Yes	36.2 (162)	33.5 (85)	56.3 (73)	χ²=18.23
	No	50.5 (226)	66.5 (169)	43.7 (55)	df=1
					p=0.00
How well do you believe that measures have been implemented to protect staff from tobacco smoke?	1 – Very well	6.9 (31)	4.2 (12)	9.2 (14)	
	2	29.9 (134)	26.3 (75)	35.5 (54)	
	3	30.6 (137)	32.6 (93)	28.9 (44)	
	4	16.1 (72)	18.6 (53)	12.5 (19)	
	5 – Very poorly	9.8 (44)	11.6 (33)	7.2 (11)	

**Table 3:** Attitudes towards specific measures to create a smoke-free environment in participating members of staff in eight psychiatric units, Berlin, Germany, 2019 (N=448)

*Question*	*Reply*	*Total % (n)*	*Current non-smokers % (n)*	*Current smokers % (n)*	*Comparison statistics*
**Which of the following measures do you support?**					
Providing self-help material for employees who smoke	Strongly favor	56.7 (254)	62.1 (177)	46.7 (71)	
	Somewhat favor	35.3 (158)	30.9 (88)	42.8 (65)	
	Somewhat oppose	4.2 (19)	2.8 (8)	7.2 (11)	
	Strongly oppose	0.9 (4)	0.7 (2)	1.3 (2)	
Therapeutic interventions for employees to help them quit smoking (individual sessions, groups)	Strongly favor	57.4 (257)	62.8 (179)	46.7 (71)	
	Somewhat favor	29.7 (133)	25.6 (73)	37.5 (57)	
	Somewhat oppose	8.7 (39)	6.7 (19)	12.5 (19)	
	Strongly oppose	0.9 (4)	0.7 (2)	1.3 (2)	
Providing nicotine replacement therapy (nicotine patches, gum, etc.) for employees who want to quit smoking	Strongly favor	38.2 (171)	35.4 (101)	42.1 (64)	
	Somewhat favor	31.3 (140)	28.8 (82)	36.8 (56)	
	Somewhat oppose	19.4 (87)	22.8 (65)	13.2 (20)	
	Strongly oppose	6.0 (27)	6.0 (17)	5.9 (9)	
Providing medication (varenicline, bupropion, etc.) for employees who want to quit smoking	Strongly favor	19.2 (86)	15.8 (45)	25.0 (38)	
	Somewhat favor	17.2 (77)	16.1 (46)	19.7 (30)	
	Somewhat oppose	37.5 (168)	36.5 (104)	39.5 (60)	
	Strongly oppose	16.5 (74)	17.5 (50)	13.8 (21)	
Special events to provide information on smoke-free hospital environments	Strongly favor	41.3 (185)	48.8 (139)	27.6 (42)	
	Somewhat favor	36.8 (165)	37.9 (108)	34.9 (53)	
	Somewhat oppose	12.3 (55)	7.4 (21)	22.4 (34)	
	Strongly oppose	4.2 (19)	1.4 (4)	7.9 (12)	
Financial incentives for smokers who successfully quit	Strongly favor	23.7 (106)	16.8 (48)	36.2 (55)	
	Somewhat favor	19.9 (89)	16.5 (47)	26.3 (40)	
	Somewhat oppose	25.2 (113)	29.5 (84)	18.4 (28)	
	Strongly oppose	25 (112)	29.5 (84)	15.1 (23)	
Additional day of annual leave for non-smokers	Strongly favor	41.5 (186)	53 (151)	22.4 (34)	
	Somewhat favor	13.4 (60)	13.3 (38)	13.8 (21)	
	Somewhat oppose	19.0 (85)	15.4 (44)	25.7 (39)	
	Strongly oppose	21.0 (94)	13.7 (39)	31.6 (48)	
Additional smoking bans on hospital premises (e.g. abolition of designated smoking areas)	Strongly favor	10.0 (45)	14.7 (42)	2.0 (3)	
	Somewhat favor	10.3 (46)	14.0 (40)	3.9 (6)	
	Somewhat oppose	36.2 (162)	38.9 (111)	32.9 (50)	
	Strongly oppose	36.4 (163)	22.8 (65)	57.9 (88)	
Penalties or disciplinary consequences for violations of non-smoking regulations	Strongly favor	8.3 (37)	11.6 (33)	2.6 (4)	
	Somewhat favor	17.2 (77)	20.4 (58)	10.5 (16)	
	Somewhat oppose	33.7 (151)	33.3 (95)	35.5 (54)	
	Strongly oppose	33.7 (151)	25.6 (73)	48.0 (73)	
**Which areas on the hospital grounds do you think should be smoke-free?**	All without exception	11.4 (51)	16.1 (46)	3.3 (5)	
	All with the exception of designated smoking areas	81.7 (366)	80.0 (228)	84.2 (128)	
	Smoking should be permitted anywhere outside the buildings	5.6 (25)	2.8 (8)	10.5 (16)	
	Smoking should be allowed everywhere	0.5 (2)	0 (0)	1.3 (2)	
**Are you worried that the implementation of further non-smoking regulations will put an additional burden on you?**	Yes	33.3 (149)	21.2 (58)	57.1 (84)	χ²=55.02
	No	62.5 (280)	78.8 (215)	42.9 (63)	df=1
					p=0.00
**Will staff work more efficiently if there are further restrictions in relation to smoking?**	Yes	27.5 (123)	42.3 (104)	13.4 (19)	χ²=34.72
	No	61.6 (276)	57.7 (142)	86.6 (123)	df=1
					p=0.00

Approximately a third of all staff members who participated in the survey felt bothered by people who smoke in the workplace environment, with non-smokers feeling significantly more bothered than smokers (χ^2^=71.45; df=1; p=0.00) (Table 2). Two in three participants believed that medical and therapeutic staff serve as role models when it comes to smoking, with significantly more non-smokers holding this view (χ^2^=38.12; df=1; p=0.00). A third thought that staff should be allowed to smoke in the presence of patients, with smokers expressing this opinion significantly more often (χ^2^=18.23; df=1; p=0.00).

Four in five participants were happy with the current regulations that smoking is only allowed in designated smoking areas; 11.4% supported a complete smoking ban. Two-thirds did not worry that the implementation of more restrictive non-smoking regulations would put a burden on them, but smokers were significantly more concerned in this regard than non-smokers (χ^2^=55.02; df=1; p=0.00). One in three participants expected that staff would work more efficiently if there were fewer opportunities to smoke in the workplace environment. Non-smokers (42.3%) expressed this view significantly more often than smokers (13.4%) (χ^2^=34.72; df=1; p=0.00).

A vast majority expressed agreement with non-restrictive measures such as organizing special events to give out information on smoke-free environments (78.1% in favor) or providing therapeutic interventions for staff members who smoke like self-help material (91.9% in favor), individual support or group sessions (87.0% in favor) or the provision of nicotine replacement therapy (69.4% in favor).

Opinions were divided when asked about financial incentives for smokers if they successfully quit (43.5% in favor vs 50.2% opposed). Staff were similarly divided on the idea to grant an additional day of annual leave for non-smokers (54.91% in favor vs 40% opposed), a model implemented by the privately owned Helios Hospital Group which runs 86 hospitals in Germany^26^. More restrictive measures such as additional smoking bans on hospital premises (72.5% opposed) and penalties or disciplinary measures for violations of non-smoking regulations (67.4% opposed) did not find support amongst staff. Interestingly, a significant proportion (19.2%) of staff who smoke stated that additional smoking bans on premises or disciplinary measures for violations would actually make them more likely to smoke.

Current smokers were asked further questions about their smoking habits (Table 4) and what effect possible measures would have on their own smoking habits (Table 5). The vast majority of daily and occasional smokers smoked cigarettes (95% vs 94.3%) and about one in ten used new electronic nicotine delivery systems (12.1% vs 11.3%). When asked about their motivation and confidence to quit on a Likert scale (1=very high to 5=very low), daily smokers rated their motivation to quit smoking with an average score of 3.37 ± 1.05 and occasional smokers with an average of 2.84 ± 0.99, while daily smokers assessed their confidence with an average of 3.03 ± 1.11 and occasional smokers with an average of 2.06 ± 0.95. Daily smokers were more interested in having smoking cessation support from their employer (38.4%) than occasional smokers (24.5%).

**Table 4:** Smoking habits of current daily and occasional smokers in participating members of staff in eight psychiatric units, Berlin, Germany, 2019 (N=152)

*Question*	*Total of daily smokers % (n)*	*Total of occasional Smokers % (n)*
**Do/did you smoke?**		
Cigarettes	95.0 (94)	94.3 (50)
Cigars	1.0 (1)	3.8 (2)
Pipes	1.0 (1)	1.9 (1)
E-cigarettes	12.1 (12)	11.3 (6)
Shisha	2.0 (2)	3.8 (2)
Other	3.0 (3)	0 (0)
**Smoking frequency**		
≤10 per day	45.5 (45)	
11–20	39.4 (39)	
21–30	9.1 (9)	
≥31	2.0 (2)	
Weekly		50.9 (27)
Monthly		24.5 (13)
Less often		20.8 (11)
**How do you rate your motivation to quit smoking?**		
1 – Very high	4.0 (4)	7.6 (4)
2	12.1 (12)	24.5 (13)
3	45.5 (45)	47.2 (25)
4	18.2 (18)	9.4 (5)
5 – Very low	19.2 (19)	7.6 (4)
**How do you rate your confidence that you could succeed in quitting?**		
1 – Very high	7.1 (7)	30.2 (16)
2	24.2 (24)	32.1 (17)
3	36.4 (36)	26.4 (14)
4	17.2 (17)	1.9 (1)
5 – Very low	12.1 (12)	1.9 (1)
**Would you like to have more support by your employer to quit smoking?**		
Yes	38.4 (38)	24.5 (13)
No	49.5 (49)	67.9 (36)

**Table 5:** Possible effect of measures to create a smoke-free environment on smoking habits of daily smokers in participating members of staff in eight psychiatric units, Berlin, Germany, 2019 (N=99)

*How would the following measures affect your smoking habits?*	*Reply*	*Total of current daily Smokers % (n)*
Providing self-help material for employees who smoke	More likely to quit	3.0 (3)
	More likely to smoke less	22.2 (22)
	No effect	68.7 (68)
	More likely to smoke more	2.0 (2)
Therapeutic interventions for employees to help them quit smoking (individual sessions, groups)	More likely to quit	25.3 (25)
	More likely to smoke less	24.2 (24)
	No effect	43.4 (43)
	More likely to smoke more	3.0 (3)
Providing nicotine replacement therapy (nicotine patches, gum, etc.) for employees who want to quit smoking	More likely to quit	24.2 (24)
	More likely to smoke less	36.4 (36)
	No effect	34.3 (34)
	More likely to smoke more	3.0 (3)
Providing medication (varenicline, bupropion, etc.) for employees who want to quit smoking	More likely to quit	21.2 (21)
	More likely to smoke less	15.2 (15)
	No effect	44.4 (44)
	More likely to smoke more	6.1 (6)
Special events to provide information on smoke-free hospital environments	More likely to quit	2.0 (2)
	More likely to smoke less	20.2 (20)
	No effect	62.6 (62)
	More likely to smoke more	8.1 (8)
Financial incentives for smokers who successfully quit	More likely to quit	33.3 (33)
	More likely to smoke less	23.2 (23)
	No effect	35.4 (35)
	More likely to smoke more	4.04 (4)
Additional day of annual leave for non-smokers	More likely to quit	21.2 (21)
	More likely to smoke less	17.2 (17)
	No effect	39.4 (39)
	More likely to smoke more	13.1 (13)
Additional smoking bans on hospital premises (e.g. abolition of designated smoking areas)	More likely to quit	1.0 (1)
	More likely to smoke less	13.1 (13)
	No effect	51.5 (51)
	More likely to smoke more	19.2 (19)
Penalties or disciplinary consequences for violations of non-smoking regulations	More likely to quit	2.0 (2)
	More likely to smoke less	11.1 (11)
	No effect	46.5 (46)
	More likely to smoke more	19.2 (19)

When asked about possible effects of specific measures on their own smoking habits, respondents deemed non-restrictive measures focusing on providing information (self-help material, organizing events) not to affect their smoking status, whereas a majority saw providing therapeutic support or nicotine replacement therapy as possibly helpful. Interestingly, financial incentives or extra annual leave was seen as likely to have an effect on their smoking habits, even though opinions of all participants towards these measures were divided. Additional restrictions or disciplinary measures were seen as counterproductive.

## DISCUSSION

With its demographic data, the sample lies close to the German average with a slightly lower percentage of daily smokers (22.1% vs 22.5%), but a higher percentage of occasional smokers (11.8% vs 5.9%) than in the general public. The higher proportion of smokers in men than in women also corresponds with data on the population in Germany as a whole^27^. The higher prevalence of smokers amongst nursing staff in comparison with other professional groups has already been reported in the literature in other countries^28^. The authors discuss that gender, income and socioeconomic differences may play a role. Higher smoking prevalence amongst staff in substance-abuse services has also been reported before^29,30^.

The results show certain contradictions in staff attitudes toward smoking prevention in psychiatric hospitals. Even though a majority of employees believe that staff serve as role models and should not smoke in the presence of patients, most participants do not see the need to further restrict the comparatively ample opportunities for staff and patients to smoke in the hospital. Significant differences in this respect can be found between smokers and non-smokers. Non-smokers generally expressed more dissatisfaction with the *status quo* and see a need for change.

When it comes to the attitude towards specific measures to create a smoke-free environment, the participants seemed to support non-restrictive measures like organizing events and offering therapeutic support, nicotine replacement therapy and information. Measures such as a special bonus or additional annual leave, did not meet general support of staff, possibly because they provide incentives to non-smokers which are denied to staff who smoke. Even though a number of current smokers state that financial incentives or an additional day of annual leave could potentially motivate them to quit smoking, the opinions of all participants on these measures are generally divided. More restrictive measures such as penalties or disciplinary action against smoking were met with overwhelming opposition. This picture of staff showing less support for more restrictive measures is even more pronounced in staff who smoke. Some of them even state that these could cause them to smoke more rather than less, which could be interpreted as an act of resistance against interventions which they regard as too harsh.

These results show that individual smoking status plays a significant role in the attitudes towards implementing measures to create a smoke-free environment. This can also be seen in the behavior of participants to answer the survey questions. For those participants, who abandoned answering the questionnaire, Lime Survey provides information at what point they stopped answering questions. For many, this seemed to be the case when asked about demographics and smoking habits. It is possible that these employees had concerns about data protection despite clear information on the anonymity of the data provided at the start. This supports the view that individual factors like smoking status are likely to be relevant factors in staff attitudes towards a smoke-free environment.

The data clearly show that staff who smoke show significantly more resistance against implementing measures to create a smoke-free environment in psychiatric hospitals. In the survey, staff who smoke were asked if they believed that the measures to create a smoke-free hospital could also influence them in their private smoking behavior. Results show that they mostly supported only those measures which they believe are unlikely to have an effect on their own smoking status. At the same time, the majority of staff who smoke accepted that they serve as role models for patients. It is possible that staff who smoke see themselves as different from their patients, which could explain the discrepancy between the measures they support for their patients and the measures which would have an effect on themselves. Another possible explanation is that their attitude towards specific measures is shaped by what they suppose would make their life in the workplace more difficult, which explains the high number of smokers saying that they worry that more restrictive regulations will place a burden on them.

These contradictory views could be the starting point for eliciting a shift in attitude in psychiatric staff who smoke. It should be taken into account that evidence shows that smoking restrictions have an effect on people’s attitudes and that staff often change their views after a complete smoking ban is introduced^31,32^. But staff resistance against measures to create smoke-free hospital environments can also be overcome through staff training schemes. Training health professionals in smoking cessation has been found to be cost-effective and to have a measurable effect in performance^33,34^. The conflicting views of staff who smoke could be a target when providing staff training on smoking cessation in psychiatry. Motivational interviewing is an approach which explores ambivalence to encourage behavior change^35^. The psychological distress caused by contradictory information or views can serve as the focus of an intervention^36^.

### Limitations

The data collected can only provide information on association, not causation. As demographic data on psychiatric staff as a whole were not available, it is not possible to generalize the findings to *Vivantes* psychiatric staff as a whole. The response rate was higher in psychiatric units with a designated task force on smoke-free service provision, which may be indicative of selection bias. It is also possible that smokers were less likely to participate as they may have been reluctant to answer questions about their smoking status.

## CONCLUSIONS

In comparison with the rest of Europe, Germany falls far behind when it comes to implementing smoke-free environments in healthcare settings. Even though staff in psychiatric units in Berlin show general support for a largely smoke-free environment, most are critical about a complete smoking ban. Staff who smoke show a significantly higher tendency to resist measures to create smoke-free environments and they tend to support only those measures which they deem unlikely to have an effect on their own smoking behavior. The contradictory views of staff towards smoking behavior – namely acknowledging the need to reduce smoking behavior but resisting concrete measures to implement a smoke-free environment – could be a focus when providing staff training, as it might prove to be a starting point for behavior change and a shift in attitude.

## Data Availability

The data supporting this research are available from the author on reasonable request.
